# Effects of Positive Human Contact during Gestation on the Behaviour, Physiology and Reproductive Performance of Sows

**DOI:** 10.3390/ani11010214

**Published:** 2021-01-16

**Authors:** Megan E. Hayes, Lauren M. Hemsworth, Rebecca S. Morrison, Kym L. Butler, Maxine Rice, Jean-Loup Rault, Paul H. Hemsworth

**Affiliations:** 1 The Animal Welfare Science Centre, The University of Melbourne, Parkville, VIC 3010, Australia; lauren.hemsworth@unimelb.edu.au (L.M.H.); kym.butler@unimelb.edu.au (K.L.B.);mrice@unimelb.edu.au (M.R.); phh@unimelb.edu.au (P.H.H.); 2 Rivalea Australia Pty Ltd., Corowa, NSW 2646, Australia; RMorrison@rivalea.com.au; 3 Biometrics Team, Agriculture Victoria Research, Department of Jobs, Precincts and Regions, Hamilton, VIC 3300, Australia; 4 Institute of Animal Welfare Science, University of Veterinary Medicine, 1210 Vienna, Austria; Jean-Loup.Rault@vetmeduni.ac.at

**Keywords:** handling, positive human contact, stress, animal welfare, reproduction, sows

## Abstract

**Simple Summary:**

Stress can compromise animal welfare and impact the productivity of farm animals. In intensive production systems where close interactions between stockpeople and animals occur regularly, human contact of a positive nature may facilitate stress resilience. This experiment studied the effects of human interaction on stress in pigs, by providing sows in their home pens with either regular positive handling by stockpeople or routine human contact. Stress resilience was studied by assessing the behaviour, physiology and productivity of pigs in these two treatments. Positive human contact was effective at reducing the fear responses of sows towards stockpeople conducting routine husbandry practices in the home pens. However, the positive handling treatment did not affect the behaviour of pigs towards other stressors imposed outside of the home pen, acute or basal physiological measures of stress or reproductive performance. Sows receiving positive handling showed reduced physical interaction with the stockpeople delivering the treatment over time, which may indicate habituation to the novel or possible rewarding elements of the human contact treatment. This work confirms that regular positive interaction with stockpeople does reduce the fear of sows to stockpeople, but does not always confer stress resilence.

**Abstract:**

Previous positive interactions with humans may ameliorate the stress response of farm animals to aversive routine practices such as painful or stressful procedures, particularly those associated with stockpeople. We studied the effects of positive handling by providing younger (parity 1–2) and older (parity 3–8) sows housed in pens of fifteen (*n* = 24 pens in total) with either positive human contact (+HC) or routine human contact (control) during gestation. The +HC treatment involved a familiar stockperson patting and scratching sows and was imposed at a pen-level for 2 min daily. Measurements studied included behavioural, physiological and productivity variables. The +HC sows showed reduced avoidance of the stockperson conducting pregnancy testing and vaccination in the home pens, however the behavioural and cortisol responses of sows in a standard unfamiliar human approach test did not differ. There were no effects of +HC on aggression between sows, serum cortisol or serum brain-derived neurotrophic factor concentrations during gestation, or on the behavioural and cortisol response to being moved to farrowing crates. There were also no effects of +HC on the maternal responsiveness of sows, farrowing rate or the number of piglets born alive, stillborn or weaned. Sows in the +HC pens reduced their physical interaction with the stockpeople imposing the treatment after 2 weeks, which suggests the sows may have habituated to the novel or possible rewarding elements of the handling treatment. This experiment shows that regular positive interaction with stockpeople does reduce sows’ fear of stockpeople, but does not always confer stress resilence.

## 1. Introduction

Stress, which refers to the biological response of an individual to an environmental change, can threaten animal welfare and compromise the productivity of farmed animals [1]. The degree to which animal welfare and productivity are affected by stress is dependent on the strength of the stressor, and how resilient the animal is to stress. In animal production systems where husbandry and housing practices involve close human contact, human interaction of a positive nature may facilitate stress resilience [2]. For example, previous positive handling reduced heart rate in calves during transport and slaughter [3], and reduced kicking and restless behaviour in dairy cows during rectal palpation [4]. Stroking in the first 2 weeks of life increased daily weight gain in calves from birth until weaning [5], and slow predictable movements and stroking during handling improved fertility rates in hens [6] and enhanced the expression of maternal care in dairy goats postpartum [7].

The majority of studies assessing the impact of handling on pigs have demonstrated that frequent negative interactions, such as shouting, slapping and hitting, increase pigs’ fear of humans and can induce both acute and chronic stress. Reduced growth, feed conversion efficiency and reproduction have all been reported in pigs as a consequence of aversive handling [8]. Although stockpeople can be associated with positive experiences (e.g., feeding [9]), most interactions between animals and stockpeople in commercial production systems are inadvertently negative for pigs. Almost all husbandry practices, that can elicit stress and pain in animals, are imposed by humans. However, positive interactions with humans may ameliorate the aversiveness of many husbandry practices, including those involving surgical interventions, given that handling *per se* may be just as stressful as the pain associated with the procedure [10]. For example, piglets that were patted and stroked during suckling bouts on the first day of life showed shorter durations of escape behaviour during tail docking at 2 days of age and capture at 15 days of age, in comparison to non-handled piglets [11].

Few other studies have researched the effects of additional positive human interaction for pigs relative to minimal or routine handling. Positive handling (talking to and patting sows when they approached) for 3 min per day reduced the magnitude of the physiological stress response to tether housing in sows [12]. Talking to, stroking and rubbing sows for 1 min per day improved ease of handling and reduced fear of humans, farrowing duration and the occurrence of piglets being savaged [13]. Though not all of the differences in the latter study were statistically significant, the authors report that the trends from all variables measured were consistent in terms of indicating there were benefits from the positive contact treatment. De Meyer et al. [14] reported reduced piglet mortality by providing sows with daily back scratching and music. Anderson et al. [15] found no effects of positive handling on piglet survival or early piglet weight gain; however, positive handling resulted in shorter durations of farrowing for sows which had high levels of fear towards humans at the start of the study.

Furthermore, there is evidence that stroking and talking softly induces an optimistic cognitive bias in piglets [16], and stimulates the release of oxytocin, a hormone most often associated with positive social behaviour, in weaner pigs [17]. These findings, in addition to the ones above, indicate that positive human contact has the potential to mitigate deleterious stress effects that pigs may encounter in commercial environments, but also confer broader stress resilience by offering stimulation in an otherwise barren environment. Stress resilience can be measured by the behavioural and physiological responses of pigs to stressors [18]. Brain-derived neurotrophic factor (BDNF), a neurotrophin which plays an important role in neuroplasticity, is also thought to mediate stress resilience by regulating HPA activity [19], and is being increasingly studied in farm animals [20,21]. An individual’s response to a stressor is influenced by the animal’s previous experience with stress [1], and thus age and/or experience may impact stress resilience. The aim of this experiment was therefore to examine the effects of regular positive human contact during gestation on stress resilience to routine management practices of sows from two different parity groups. 

## 2. Materials and Methods

### 2.1. Animals and Housing

This experiment was conducted at a large commercial piggery in southern New South Wales, Australia. All animal procedures were conducted with prior institutional ethical approval under the requirements of the New South Wales Prevention of Cruelty to Animals Act 1985 in accordance with the National Health and Medical Research Council/Commonwealth Scientific and Industrial Research Organization/Australian Animal Commission *Code of Practice for the Care and Use of Animals for Scientific Purposes.*

Three hundred and sixty mixed-parity Landrace × Large White sows that were in good health at the start of the experiment were studied throughout gestation (treatment period) and lactation. Sows were studied over two-time replicates, conducted in late spring and late winter, and were housed in groups of fifteen (*n* = 24 pens) with sows of the same parity group (parity 1–2 or parity 3–8) during gestation. Within each time replicate, half of the sows were introduced to the experiment in week 1 of the experiment while the remaining half were introduced to the experiment in week 2. In each of these weeks, 90 sows (45 parity 1–2 and 45 parity 3–8) which had been inseminated within the previous 4 days were selected and assigned to the gestation pens that had been allocated to the appropriate parity (see subsection section “Design” for further detail). There were two adjacent rows of partially slatted floored pens (6 pens/row) separated by a central corridor, each with a space allowance of 2 m^2^/sow. Overhead water sprinklers covering 50% of each pen were activated when the internal temperature exceeded 26 °C. Feed (2.5 kg/sow/day of a commercial diet; 13.1 MJ/kg DM, and 13.5% CP) was delivered onto the solid section of the floor via automatic overhead drop feeders (5/pen) twice per day (approximately 07:00 and 07:30 h). The shed was renovated for the experiment, so that all pens had solid sides to limit visual contact between adjacent pens; however, the gates on the front of the pens were not solid, allowing visual contact with people and pigs in and across the corridor. Two CCTV cameras were placed over each pen. Sows were introduced to farrowing crates (2.3 × 1.7 m) at least 5 days prior to expected parturition, with sows from the same gestation pen generally being in nearby farrowing crates. Fostering piglets within treatment was performed when necessary. No bedding or nesting material was provided during the gestation or farrowing/lactation periods.

### 2.2. Treatments

A factorial treatment design with the following main effects was used to study the effects of positive human contact and age/experience on sow behaviour, physiology and reproductive performance:

1. Human contact
  i. *‘Positive human contact (+HC)’*—At 13:00 h daily, one of five male stockpeople trained to impose this handling treatment entered each +HC treatment pen for 2 min, walked slowly through the group and stopped to pat, stroke, scratch and talk softly to sows in their pathway and sows approaching. The +HC treatment was imposed from post-insemination mixing until sows were moved to farrowing accommodation in week 16 of treatment. The stockpeople imposing this handling treatment were responsible for routine management of all sows.
  ii. *‘Control contact’*—this treatment involved human contact only associated with routine management. Routine management of all sows involved twice daily health and welfare checks by the same stockpeople responsible for imposing the +HC treatment. These welfare checks involved one stockperson visually inspecting the animals and the facilities. Welfare checks generally took less than 30 s per pen and were usually conducted in the aisle, although sometimes it was necessary for stockpeople to enter the pen.
2. Parity
  i. *‘Younger’*—Parity 1–2 sows
  ii. *‘Older’*—Parity 3–8 sows

### 2.3. Design

Within each row the pens were assigned to three pairs of adjacent, or nearly adjacent, pens. In each of these pairs one pen had younger sows and the other had older sows. Additionally, in each of these pairs, one pen received the +HC treatment and the other pen received the control contact treatment. Thus, each pair of pens had one pen of younger sows with +HC and one pen of older sows with control contact (Pair combination A) or alternatively had one pen of younger sows with control contact and one pen of older sows with +HC (Pair combination B). Within each of the two time-replicates, there was one row with two pairs of pair combination A and one pair of pair combination B, and the other row had one pair of pair combination A and two pairs of pair combination B. Within these restrictions, the combinations of human contact treatment and sow parity group were assigned randomly to the 24 pens. The ANOVA structure of this design is presented in Table 1.

### 2.4. Measurements

#### 2.4.1. Sow and Stockperson Behaviour during +HC Treatment Imposition

Using CCTV camera footage, sow and stockperson behaviour was recorded during the imposition of +HC on days 2, 5, 7, 9, 11, 15, 17, 28, 43, 58 and 73 of treatment. Measures of stockperson behaviour included the proportion of sows in each +HC pen the stockperson physically interacted with in terms of patting, stroking or scratching a sow, and the total number of tactile interactions initiated by stockpeople. Measures of sow behaviour included the total number of tactile interactions with the stockperson (sniffing, nosing or chewing the stockperson or their attire) and the mean proportion of sows in each +HC pen within 1 m of the stockperson (recorded using instantaneous scan sampling every 10 s during the 2 min treatment imposition). 

#### 2.4.2. Responses towards Routine Management and Husbandry Practices

All sows underwent routine husbandry and management practices imposed in commercial production. This included pregnancy testing with an external ultrasound probe (week 6 of treatment), intramuscular vaccination (week 12 of treatment) and being moved to farrowing accommodation (end of treatment, week 16). During the pregnancy testing and vaccination that were conducted in the home pens, CCTV footage was used to record the approach–withdrawal responses of sows to the stockperson. When the approaching stockperson was within 1 m of a sow before conducting pregnancy check and vaccination, the sow was recorded as either withdrawing (moved away from the stockperson within 2 s) or not withdrawing (approached the stockperson or remained stationary). The response of each sow was recorded again immediately after (within 2 s) pregnancy check and vaccination had been conducted. The number of sow-initiated tactile interactions was also recorded, but only during pregnancy testing as the stockperson was holding a stockboard during vaccination which prevented sows from physically interacting. Interactions during pregnancy testing were defined as any tactile contact including sniffing, nosing or chewing the stockperson or their attire. The stockpeople conducting pregnancy testing and vaccination were different for each husbandry practice and different between time replicates. However, the stockperson was always one of the people responsible for routine management of all pigs, and thus was familiar to both +HC and control sows. Additionally, the stockperson was also one of the five people responsible for imposing the +HC treatment. When sows were being moved into farrowing accommodation, direct observations were used to record the behaviour of sows while entering the farrowing crates. When each sow reached within 1 m of the crate, they were recorded as either voluntarily entering (walked into the crate without stopping or turning in the aisle) or resisting entry (attempted to turn around or stopped for more than 2 s in the aisle, requiring the stockperson to use pushing or slapping to move the sow into the crate). At 1.5, 3.5 and 4.5 h after introduction to the farrowing crates, the behaviour of 6 randomly selected sows from each gestation pen was observed. For periods of 30 s at each of the three time points, one-zero sampling was used to record whether or not sows were lying, bar biting (chewing/mouthing crate fittings), sham chewing (repetitive chewing in the absence of feed) or vocalising. Saliva samples for subsequent analysis of cortisol were collected from 6 randomly selected sows from each gestation pen 2.5 h after introduction to farrowing crates (see subsequent section “Physiological sample collection details and assay characteristics” for further detail). Four weeks after housing in the farrowing crates, saliva samples for subsequent analysis of cortisol were collected again from another 6 sows randomly selected from each gestation pen, as previous research has demonstrated that cortisol concentrations in gilts housed in farrowing crates increase at 4 weeks postpartum relative to gilts in lactation pens [22].

#### 2.4.3. Responses towards Humans in a Standard Human Approach Test

The behavioural response to an unfamiliar stationary human in a novel arena was assessed in 6 sows randomly selected from each pen during week 9 of treatment, following the method described by Hemsworth et al. [23]. The human approach test (HAT) arena (3 × 3.8 m) was constructed with solid black boarding within the shed where sows were housed. Painted lines were used to mark quadrants on the floor of the arena as well as a 0.5 m radius half-circle on the floor adjacent to the mid-point of the wall opposite the arena entry, in which the human stood. Testing was undertaken from 08:30 to 15:00 h, starting at least 1 h after feeding. Prior to testing, the 6 test sows from the first pen were moved into a holding area located 9 m away from the test arena. These 6 sows were given 4 min to settle in the holding area, before the first test sow was removed from the group and individually introduced to the empty test arena for a familiarisation period of 2 min. During the familiarisation period, the number of entries into quadrants was recorded as a measure of exploration in the novel arena. At the end of the familiarisation period, the human slowly entered and walked to the marked mid-point of the wall. The human stood stationary in this position for 3 min, and verbally relayed the sow’s behaviour to an assistant standing outside the arena who was not visible to the sow, to record the following behavioural variables: latency to approach within 0.5 m of the human, time spent within 0.5 m of the human, latency to interact and number of tactile interactions with the human. Interactions were defined as any tactile contact including sniffing, nosing or chewing the human or their attire, and a bout criterion of 5 s was chosen to separate one bout of interaction from another bout. Upon completion of the test, the human collected a saliva sample from the sow for later analysis of cortisol (see subsequent section “Physiological sample collection details and assay characteristics” for further detail). The test sow was then moved back to the holding pen and the next sow from the group was walked into the arena. Once all sows from the group had been tested, they were moved back to the home pen and the 6 test sows in the next pen along the aisle were moved to the holding area. All sows were moved by experienced handlers, unfamiliar to all pigs, with the assistance of a solid pig board.

#### 2.4.4. Aggressive Behaviour between Sows during Feeding

Using CCTV camera footage, aggression during feeding was sampled for 1 day in weeks 1, 4 and 8 of treatment. Aggressive behaviour was defined as slashes, butts, pushes, and bites, and these were distinguished from other tactile interactions with sows on the basis that the former were associated with avoidance or retaliation by one sow as a consequence of the interaction. The number of aggressive interactions between sows in each pen was recorded continuously by one observer for 15 min after each of two morning feed drops, spaced 30 min apart. This time period was chosen as aggression between sows during feeding is most intense in the first 15 min after feed is dropped [24]. Only aggressive interactions in which the head of the sow (defined as extending from the snout to the ears) displaying the aggressive behaviour was clearly visible were recorded, and so the number of aggressive interactions in each pen was expressed based on the average number of sows visible in the camera’s field of view. For each 15 min sampling period, instantaneous scan sampling at 30 s intervals was used to count the number of sows in the field of view. Aggression in each pen was then calculated by multiplying the number of aggressive interactions by the inverse of the average number of sows in the field of view. 

#### 2.4.5. Basal Physiological Measurements during Gestation

In weeks 5 and 10 of treatment, blood samples were collected from 3 sows randomly selected from each pen for later analysis of serum cortisol and serum brain-derived neurotrophic factor (BDNF) (see subsequent section “Physiological sample collection details and assay characteristics” for further detail). 

#### 2.4.6. Maternal Responsiveness Test

In week 2 of lactation (i.e., 3 weeks after treatment had finished), the maternal behaviour of all sows was assessed by recording their behavioural responses towards an audio recording of an unfamiliar piglet squealing. Following the protocol for the maternal responsiveness test as described by Singh et al. [25], an experimenter wearing a portable stereo unit around their neck entered the farrowing shed 5 min after sows had received their first feed delivery for the day (approximately 8:00 h). The experimenter then turned on the stereo unit and broadcasted a pre-recorded sound of a squealing piglet (80 decibels at a distance of 1 m) while walking slowly through the farrowing shed, pausing for 3 s in front of each sow’s crate. The experimenter repeated the test six times, with each test taking approximately 10 min to complete. Two Go-Pro cameras were mounted on either side of the experimenter’s head, which allowed the sows’ behavioural responses during each 3 s bout of the audio broadcasting to later be assessed by one observer. Maternal responsiveness in the test was measured through changes in posture (changed from lying or sitting to standing), disruptions to feeding (lifted head from feeder) and the occurrence of sows vocalising, displaying behaviour directed towards their piglets (turned head to look at, sniff or nose piglets) and bar biting (chewing or mouthing crate fittings). 

#### 2.4.7. Reproductive Performance

Records of farrowing rate (proportion of sows inseminated that farrowed), the total number of piglets born alive, stillborn and mummified and the total number of piglets weaned were collected for all sows. 

#### 2.4.8. Physiological Sample Collection Details and Assay Characteristics

A random number generator was used to select sows from each pen for the collection of physiological samples. Saliva and blood samples were all collected within 2 min of approaching the sow, and in the event that a sow could not be sampled within this period, another sow from the pen was chosen. All samples were collected between 10:00 and 12:00 h, with the exception of post-HAT saliva samples which were obtained after each sow had been tested. For collection of saliva, 2 experimenters collected samples from sows in the pen simultaneously. A synthetic swab (Salimetrics; Carlsbad, California, USA) was secured to the end of a cable tie and offered to the sow to chew on for 1–2 min or until it was suitably moistened. The swab was then placed into a saliva collection tube (Sarstedt; South Australia, Australia) and kept on ice before being centrifuged at 2500× *g* for 2 min. After centrifugation, saliva was pipetted into polypropylene tubes and stored at −20 °C until later analysis of cortisol. For collection of blood, 2 experimenters were used for each sow; the first restrained the sow with a snout snare and the second collected the blood sample. Samples were obtained in 6 mL serum collection tubes (BD Vacutainer, New South Wales, Australia) via jugular venepuncture. After collection, tubes were gently inverted five times and allowed to clot for 1 h at room temperature. Samples were then centrifuged at 1000× *g* for 15 min, and the designated serum was pipetted off into two polypropylene tubes and stored at −20 °C until later analysis of cortisol and BDNF. Salivary and serum cortisol were quantified using commercially available RIA kits from MP Biomedicals (Item #07221105). The intra- and inter-assay co-efficient of variations for cortisol assays were all between 2.9 and 4.2%. Serum BDNF was analysed at 1:25 dilution and quantified using a commercially available ELISA kit from Biosensit (Item #BEK-2211). The mean intra- and inter-assay coefficient of variations were 2.9 and 7.8%, respectively.

### 2.5. Statistical Analysis

The unit of analysis for all statistical analyses was the group of sows from a single pen. For measurements taken on individual sows, pen values were calculated as averages of each individual sow measurement, or as a proportion of sows with a particular response. Prior to analysis, the number of sow-initiated tactile interactions during pregnancy testing, aggressive interactions in weeks 1, 4 and 8 of treatment and serum cortisol and BDNF concentrations in weeks 5 and 10 of treatment were logarithmically transformed on a pen basis, so that the amount of residual variation did not increase as the mean increased and/or so that the residuals did not have a markedly skewed distribution. There were no BDNF concentration measurements available for any sows in two pens in week 5 and one pen in week 10, due to blood samples being haemolysed or returning results outside the normal detectable range of the assay. Thus, there were 2 missing values for BDNF at weeks 5 and 1 missing value for BDNF at week 10.

Apart from measurements of sow behaviour during +HC treatment imposition, all sow measurements were analysed using an analysis of variance of the form presented in Table 1. Human contact treatment by parity interactions were estimated in both the row within time replicate and the pair within row stratums. However, there was only a residual degree of freedom in the row within time replicate stratum and almost 90% of the structural information was estimated in the pair within row stratum (efficiency factor = 0.889; Table 1). Thus, all results presented that involve the human contact by parity interaction are only from the pair within row stratum (i.e., we used an intra-block analysis). Sow behaviour during +HC treatment measurements used a modified version of the intra-block analysis of variance that only included a treatment effect for parity and did not include a pair within row stratum. In these analyses, the effect of parity was estimated on 7 degrees of freedom. Non-parametric permutation tests were used to calculate *p*-values for the proportion of sows that were bar biting, sham chewing and vocalising after entry to farrowing crates, and the proportion of sows that were bar biting during the maternal responsiveness test. In all these cases there were many pens with no sows partaking in the behaviour. These permutation tests were calculated using the usual F statistic obtained from the analysis of variance in Table 1, but with the permutation distribution implied from the structure of this analysis of variance. Results for stockperson behaviour during +HC treatment imposition are presented as box and whisker plots profiled over the period of treatment imposition. All analyses were carried out using the ANOVA directive, the APERMTEST procedure, the AREPMEASURES procedure and the BOXPLOT procedure of GenStat for Windows 18th Edition [26].

## 3. Results

There were no significant human contact × parity interactions (*p >* 0.05) on any sow variables measured, and thus the means and standard error of differences are reported for only the main effects of human contact and parity. 

### 3.1. Sow and Stockperson Behaviour during +HC Treatment Imposition

#### 3.1.1. Stockperson Behaviour during +HC Treatment Imposition

**Figure 1:**
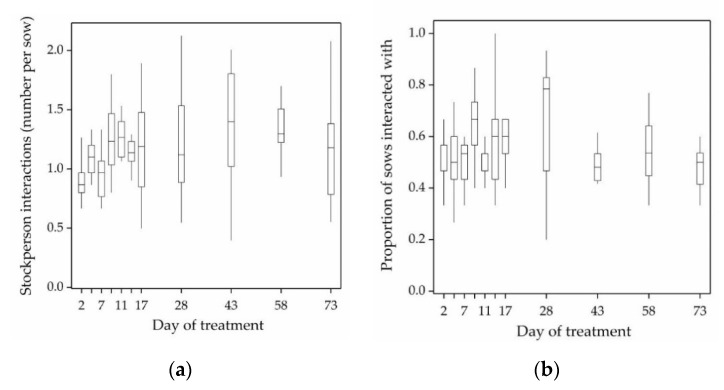
Box and whisker plots of stockperson behaviour during positive human contact imposition on days 2, 5, 7, 9, 11, 15, 17, 28, 43, 58 and 73 of treatment. 1**a** shows the number of tactile interactions (patting, stroking, scratching) by stockpeople in each +HC treatment pen. 1**b** shows the proportion of sows in each +HC pen that stockpeople interacted with.

During the 2 min treatment imposition, stockpeople interacted with an average of 54% of sows from each +HC pen and initiated 1.2 tactile interactions per sow (Figure 1a,b). 

#### 3.1.2. Sow Behaviour during +HC Treatment Imposition

**Figure 2:**
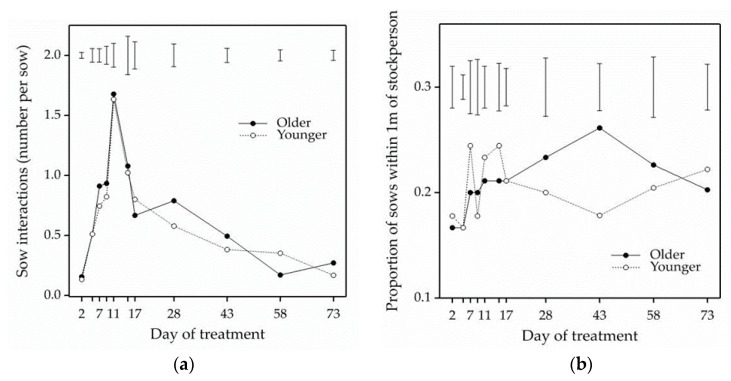
Sow behaviour during positive human contact imposition on days 2, 5, 7, 9, 11, 15, 17, 28, 43, 58 and 73 of treatment. 2**a** shows the mean number of tactile interactions (sniffing, nosing, chewing) with stockpeople initiated by older (solid line) and younger (dashed line) +HC sows. 2**b** shows the mean proportion of older (solid line) and younger (dashed line) +HC sows within 1 m of the stockperson during treatment imposition. Error bars represent standard error of the difference using single day analyses of variance, which did not require data transformation for number of sow interactions.

There were no parity effects (*p* > 0.05) on the number of tactile interactions with stockpeople initiated by +HC sows during imposition of the handling treatment. There were also no parity effects (*p* > 0.05) on the mean proportion of +HC sows within 1 m of the stockperson during treatment imposition. The number of tactile interactions by sows increased from 0.1 to 1.7 interactions per sow from day 2 until day 11 of treatment, after which the number of tactile interactions by sows reduced (Figure 2a; F_3.62,36.2_ = 38.02, *p* = 4.5 × 10^−12^ for test of day of treatment effect using a repeated measures analysis of variance with Greenhouse Geisser correction (ε = 0.3622) on square root transformed data). The mean proportion of sows from each +HC pen within 1 m of the stockperson imposing the treatment was 21% (Figure 2b; F_4.07,40.7_ = 1.13, *p* = 0.36 for test of day of treatment effect using a repeated measures analysis of variance with Greenhouse Geisser correction (ε = 0.4067)).

### 3.2. Treatment and Parity Effects

#### 3.2.1. Responses towards Routine Management and Husbandry Practices

Sows from +HC pens showed fewer withdrawal responses than sows from control pens when the stockperson approached within 1 m to conduct pregnancy testing in week 6 (12 vs. 37 % of sows; sed = 4.2; *p* < 0.001), and vaccination in week 12 of treatment (43 vs. 67 % of sows; sed = 7.2; *p* = 0.008; Table 2). There were also fewer withdrawal responses by +HC sows immediately after pregnancy testing (16 vs. 52 % of sows; sed = 3.9; *p* < 0.001) and vaccination (84 vs. 92 % of sows; sed = 3.9; *p* = 0.05). During the imposition of pregnancy testing on the entire pen, +HC sows initiated significantly more tactile interactions with the stockperson (95% confidence interval for % increase = (120, 430); *p* < 0.001). There was a higher proportion of younger sows avoiding the stockperson before (32 vs. 18 % of sows; sed = 4.2; *p* = 0.009) and after (39 vs. 29 % of sows; sed = 3.9; *p* = 0.03) the pregnancy test. However, a similar effect was not present at vaccination (*p >* 0.05). Despite good statistical precision, there were no significant effects (*p >* 0.05) of treatment or parity on the behavioural or cortisol responses of sows towards being moved from group gestation pens to individual farrowing crates. 

#### 3.2.2. Responses towards Humans in a Standard Human Approach Test

Despite good precision, there were no effects (*p >* 0.05) of treatment or parity on the latency to approach, latency to initiate tactile interaction, the total time spent in close proximity or the total number of tactile interactions with a stationary human in the HAT conducted in week 9 of treatment (Table 3). There were also no effects (*p >* 0.05) of treatment or parity on the number of quadrants entered in the 2 min familiarisation period, or on cortisol concentrations in saliva collected after the HAT.

#### 3.2.3. Aggressive Behaviour between Sows during Feeding

There were no significant effects (*p >* 0.05) of treatment or parity on the number of aggressive interactions between sows during morning feed drops in weeks 1, 4 and 8 of treatment (Table 4).

#### 3.2.4. Basal Physiological Measurements during Gestation and Lactation 

There were no significant effects (*p* > 0.05) of treatment or parity on serum cortisol or brain-derived neurotrophic factor concentrations in weeks 5 or 10 of treatment (Table 5).

#### 3.2.5. Maternal Responsiveness Test

In response to an audio recording of unfamiliar piglet squeals, there were no significant treatment or parity effects (*p >* 0.05) on the proportion of sows that moved from resting to standing, stopped feeding, vocalised, directed their behaviour towards their piglets or began biting crate fixtures (Table 6).

#### 3.2.6. Reproductive Performance

There were no effects of treatment or parity on the proportion of sows that farrowed (Table 7). There were more piglets stillborn from older parity sows compared to younger parity sows (1.4 vs. 1.0; sed = 0.14; *p* = 0.013). There were no effects (*p >* 0.05) of human contact or parity on the number of piglets born alive, mummified or weaned from each sow.

## 4. Discussion

This experiment examined the effects of regular positive human contact during gestation on stress resilience in sows from young and old parity groups. Two min of daily positive handling reduced the avoidance responses of sows to stockpeople imposing two common husbandry practices in their home pens, pregnancy check and vaccination. However, there were no effects of human contact on the behavioural and cortisol responses to the presence of an unfamiliar human or to being moved into farrowing crates, suggesting that the sows did not generalise to other humans or other contexts. Furthermore, there were no treatment effects on aggression during feeding, basal cortisol or BDNF concentrations, or the reproductive performance of the sows. 

There were fewer sows from the +HC treatment that showed avoidance of the stockperson before and after the imposition of pregnancy testing and vaccination, indicating reduced fear of these practices. It is likely these behavioural responses reflect reduced fear of the stockpeople imposing the husbandry practices, rather than reduced fear of the procedures *per se*; however, close human presence may be the most stressful element of many husbandry practices [10]. Although measuring the physiological responses towards these stressors would be valuable in further understanding the effects of positive handling on sows, these findings on the behavioural responses of sows show that previous positive interactions with stockpeople can ameliorate the stress responses of pigs towards routine husbandry practices imposed by those same persons. Reduced stress is a beneficial outcome for animal welfare, and may have additional benefits for the stockperson, as pigs which are less fearful of humans are easier to handle [13] and thus impose husbandry practices upon. Pigs with lower fear of humans may also be less likely to sustain injuries arising from avoiding stockpeople during routine inspections and handling, as demonstrated with calves [3]. There was an effect of parity with a higher proportion of younger sows withdrawing from the stockperson in comparison to older sows during pregnancy testing. These results indicate that younger sows show greater fear of humans during routine practices in commercial production systems. 

Although sows in the human contact treatment showed reduced avoidance of the stockperson imposing husbandry practices in the group pens, and despite good precision of the statistical analysis, there were no effects of +HC on the behaviour of sows when moved to and after entering farrowing crates, or on salivary cortisol concentrations after entry. There were also no effects of the handling treatment on the frequency of aggressive interactions during gestation, although aggression was only studied during feeding bouts when it is more likely to be intense due to competition between sows. To our knowledge, there have been no other studies assessing the impact of positive handling on aggression or abnormal behaviours in sows, such as sham chewing and bar biting, as observed after introduction to farrowing crates in this experiment. In weaned pigs, positive human interaction has been reported to reduce the incidence of tail biting [27], although the handling treatment in this experiment involved offering chopped straw to piglets which may have increased foraging and thus reduced tail biting behaviour. 

There were no effects of +HC on the behavioural or cortisol responses of sows towards an unfamiliar human in the HAT. Although the positive handling treatment was primarily of a tactile nature in the present experiment, interactions between pigs and stockpeople occur through various sensory channels [28]. One of the difficulties in conducting this experiment was eliminating human interaction in the control treatment that was not associated with routine management. Both control and +HC sows were allocated to one area of the gestation shed to control for positional effects in the facility. While group pens had solid sides to limit visual contact between adjacent pens, the gates on the front of the pens were not solid, and allowed visual contact with people and pigs in the aisle and in the opposite pens. The imposition of the positive handling treatment therefore unavoidably involved control sows receiving up to 30 min daily of additional visual, auditory and olfactory contact with stockpeople, which greatly exceeded the general stockperson contact in the home pen associated with routine management. Studies on poultry have shown that visual contact with an experimenter reduces birds’ fear of handling [29], and can be more effective at reducing fear of humans than gentle tactile contact [30]. Recent research has also demonstrated that pigs’ fear of humans is reduced when they observe a human stroking other pigs [31]. It is therefore possible that any treatment effects on responses in the HAT were diluted as a result of control sows receiving additional visual exposure to humans.

There are several explanations for the conflicting behavioural responses of sows to familiar stockpeople imposing the two husbandry practices and to the unfamiliar human in the HAT. It may be that a human closely approaching and imposing a husbandry procedure is more likely to differentiate pigs of varying levels of fear of humans. It is also possible that the stockperson contact control sows received during the imposition of the +HC treatment in nearby pens may have reduced the fear responses of control sows to the extent that they were similarly motivated to +HC sows to explore and interact with the human in the HAT. Furthermore, the +HC sows may not have generalised their response to unfamiliar humans in a different location. However, it should be recognised that there is evidence pigs can generalise handling experiences to other humans [32] in different locations [33], and it has been well documented that positive human contact relative to minimal handling reduces pigs’ fear of humans in a standard test such as the HAT [8,34,35]. 

A feature of the positive handling treatment that differentiates this experiment from others is that patting and stroking occurred irrespective of sows voluntarily seeking interaction from the stockperson (although if the stockperson initiated interaction and the sow retreated, the stockperson did not pursue the sow). In the majority of studies reporting benefits of positive handling, contact was “unforced”, and only occurred if pigs approached the human (e.g., reduced fear of humans [36,37]; reduced cortisol response [12]; reduced tail biting [27]; increased oxytocin [17]; and increased feed intake [38]). There is increasing attention being paid to the importance of providing animals with a sense of control over aspects of their environment, including in their interactions with humans [2]. The +HC treatment in the present experiment may have been more effective if it was contingent on pigs voluntarily soliciting interaction from the stockperson, therefore providing sows with greater choice and control in the interaction. Although the proportion of +HC sows within 1 m of the stockperson remained consistent from the start until the end of the treatment period, voluntary interaction by +HC sows with the stockpeople imposing the handling increased sharply from day 2 until day 11 of treatment, and then reduced until the end of the treatment period. While reduced interaction at the end of the treatment period may correlate with reduced activity later into gestation, it is surprising that there was a reduction in the sows’ tactile contact with the stockperson after only 2 weeks. The reduced physical interaction initiated by sows suggests habituation to any novel or reinforcing elements of the handling treatment. 

Positive human contact had no effect on the maternal behaviour or reproductive performance of sows. High levels of fear towards humans can induce chronic stress, and have been associated with an increased incidence of stillborn piglets [39], reduced pregnancy rate [36], smaller litter sizes [40], and a higher prevalence of crushing piglets [41]. There were no treatment effects on sows’ fear responses in the HAT or on basal cortisol or BDNF concentrations during gestation, and so any stress-related effects of treatment were unlikely to affect the reproductive performance of sows in this experiment. Other studies comparing the effects of positive handling of sows relative to minimal handling have reported reduced farrowing duration [15] and lower pre-weaning mortality [14]. However, farrowing duration was only reduced in highly fearful sows in the former study, and sows and piglets were also exposed to music in the latter study. In regards to the effects of parity in the present experiment, there were higher numbers of stillborn piglets from older sows than younger sows, but as reproductive performance is known to decline with age this was expected [42]. 

## 5. Conclusions

Providing group housed sows with 2 min of daily patting and scratching reduced the fear responses of pigs towards stockpeople imposing pregnancy testing and vaccination in the home pens. However, the positive handling treatment had no effect on sows’ behavioural and cortisol responses towards an unfamiliar human standing stationary, or towards being moved into farrowing crates. Aggression during feeding, basal cortisol and BDNF concentrations, and reproductive performance were not affected by the handling treatment. There was evidence that sows in the positive human contact groups habituated to the handling treatment after 2 weeks. While this experiment showed that previous positive interactions with stockpeople can ameliorate the stress responses of pigs towards routine husbandry practices, further research is necessary to understand the effects of positive handling on stress resilience when pigs have control over the interactions with humans, as well as the possible effects of habituation to positive handling.

## Figures and Tables

**Table 1:** Structure of analysis of variance, including information summary, for statistical analysis of the study.

Source of Variation	Degrees of Freedom	
*Time replicate stratum*	1	
*Row within time replicate stratum*		
Human contact treatment by parity interaction	1	
Residual	1	
*Pair within row stratum*		
Human contact treatment by parity interaction	1	
Residual	7	
*Pen within pair stratum*		
Human contact treatment	1	
Parity	1	
Residual	10	
Information summary for analysis of variance		
**Model Term**	**Efficiency factor**	**Non-orthogonal Terms**
*Row within time replicate stratum*		
Human contact treatment by parity interaction	0.111	
*Pair within row stratum*		
Human contact treatment by parity interaction	0.889	Row within time replicate

**Table 2:** Effects of human contact and parity on sows’ responses towards pregnancy testing, vaccination and being moved into farrowing crates. Means and standard error of the difference (s.e.d.) for main effects are presented. The number of tactile interactions during pregnancy testing was logarithmically (base *e*) transformed; back-transformed means are presented in parentheses.

Measurement	Human Contact		Parity			*p*-Value		
	Positive	Control	Younger	Older	s.e.d.	Human Contact	Parity	Human Contact × Parity
*Pregnancy testing*								
Proportion of sows withdrawing from the approaching stockperson	0.12	0.37	0.32	0.18	0.042	0.00016	0.0089	0.060
Proportion of sows withdrawing post pregnancy test	0.16	0.52	0.39	0.29	0.039	3.6 × 10^−6^	0.027	0.063
Tactile interactions initiated with the stockperson (number per pen)	2.8 (16)	1.6 (4.7)	2.3 (10)	2 (7.3)	0.20	0.00011	0.13	0.52
*Vaccination*								
Proportion of sows withdrawing from the approaching stockperson	0.43	0.67	0.54	0.56	0.072	0.0079	0.75	0.44
Proportion of sows withdrawing post vaccination	0.84	0.92	0.90	0.86	0.039	0.054	0.25	0.79
*Moving to farrowing crates*								
Proportion of sows resisting entry to crate	0.51	0.46	0.54	0.44	0.048	0.31	0.072	0.82
Proportion of sows lying down after entry ^1^	0.69	0.7	0.65	0.73	0.068	0.86	0.21	0.46
Proportion of sows bar biting after entry ^1^	0.06	0.06	0.04	0.07	0.022	0.84 ^2^	0.14 ^2^	1.0 ^2^
Proportion of sows sham chewing after entry ^1^	0.09	0.07	0.09	0.07	0.017	0.25 ^2^	0.33 ^2^	0.81 ^2^
Proportion of sows vocalising after entry ^1^	0.32	0.26	0.34	0.25	0.072	0.39 ^2^	0.21 ^2^	0.11 ^2^
Salivary cortisol concentrations 2.5 h after entry (ng/mL)	1.3	1.3	1.2	1.3	0.066	0.40	0.16	0.97
Salivary cortisol concentrations 4 weeks after entry (ng/mL)	1.6	1.4	1.5	1.5	0.14	0.25	0.71	0.68

^1^ Data represent the mean proportion of sows engaging in each behaviour when observed 1.5, 3.5 and 4.5 h after entry to farrowing crates. ^2^ *p*-values calculated using permutation tests.

**Table 3:** Effects of human contact and parity on sows’ responses in the human approach test. A maximum response time of 180 s was given to sows which did not approach or interact with the human. Means and standard error of the difference (s.e.d.) for main effects are presented.

Measurement	Human Contact		Parity			*p*-Value		
	Positive	Control	Younger	Older	s.e.d.	Human Contact	Parity	Human Contact × Parity
No. entries into different quadrants during the familiarisation period	11	11	11	11	0.7	0.55	0.78	0.68
Latency to approach within 0.5 m of human (s)	76	78	77	77	0.19	0.87	0.96	0.53
Time spent within 0.5 m of human (s)	28	31	33	26	0.1	0.64	0.30	0.58
Latency to physically interact with human (s)	109	108	106	111	0.19	0.98	0.66	0.65
Tactile interactions initiated with human (number per sow)	2.8	4	3.9	3	0.72	0.11	0.24	0.76
Salivary cortisol concentrations on completion of testing (ng/mL)	3.6	3.4	3.9	3.1	0.44	0.59	0.084	0.058

**Table 4:** Effects of human contact and parity on aggression between sows during feeding in the morning. Data represent the mean number of aggressive interactions per sow, based off the average number of sows in the camera’s field of view. Means and standard error of the difference (s.e.d.) for main effects are presented. Data were logarithmically (base *e*) transformed; back-transformed means are presented in parentheses.

Measurement	Human Contact		Parity			*p*-Value		
	Positive	Control	Younger	Older	s.e.d.	Human Contact	Parity	Human Contact × Parity
Aggressive interactions week 1 of treatment	1.9 (7.0)	2.1 (7.9)	2.0 (7.6)	2.0 (7.2)	0.19	0.54	0.81	0.65
Aggressive interactions week 4 of treatment	2.3 (9.5)	2.3 (9.5)	2.2 (9.3)	2.3 (9.7)	0.11	0.99	0.66	0.40
Aggressive interactions week 8 of treatment	2.0 (7.6)	2.0 (7.4)	2.1 (7.9)	2.0 (7.1)	0.12	0.84	0.30	0.44

**Table 5:** Effects of human contact and parity on basal serum cortisol and BDNF concentrations during the treatment period. Means and standard error of the difference (s.e.d.) for main effects are presented. Data were logarithmically (base *e*) transformed; back-transformed means are presented in parentheses.

Measurement	Human Contact		Parity			*p*-Value		
	Positive	Control	Younger	Older	s.e.d.	Human Contact	Parity	Human Contact × Parity
*Cortisol*								
Week 5 of treatment (ng/mL)	1.8 (5.8)	1.7 (5.4)	1.7 (5.6)	1.7 (5.6)	0.13	0.55	0.94	0.17
Week 10 of treatment (ng/mL)	2.2 (8.7)	2.3 (9.7)	2.2 (9.2)	2.2 (9.1)	0.12	0.41	0.94	0.27
*BDNF*								
Week 5 of treatment (pg/mL)	7.1 (1190)	7.3 (1420)	6.7 (830)	7.6 (2020)	0.70	0.80	0.24	0.92
Week 10 of treatment (pg/mL)	7.2 (1310)	6.8 (930)	6.6 (710)	7.5 (1720)	0.50	0.50	0.11	0.72

**Table 6:** Effects of human contact and parity on the behaviour of sows in response to an audio recording of unfamiliar piglet vocalisations. Data represent the mean proportion of sows engaging in each behaviour during the maternal responsiveness test. Means and standard error of the difference (s.e.d.) for main effects are presented.

Measurement	Human Contact		Parity			*p*-Value		
	Positive	Control	Younger	Older	s.e.d.	Human Contact	Parity	Human Contact × Parity
Changed posture from sitting or lying to upright	0.22	0.31	0.28	0.24	0.063	0.19	0.58	0.15
Disrupted from feeding	0.25	0.33	0.28	0.31	0.043	0.093	0.43	0.58
Vocalising	0.61	0.76	0.66	0.71	0.069	0.06	0.42	0.40
Behaviour towards piglets	0.39	0.40	0.36	0.43	0.068	0.98	0.29	0.98
Bar biting	0.16	0.08	0.13	0.11	0.052	0.12 ^1^	0.68 ^1^	0.06 ^1^

^1^*p*-values calculated using permutation tests.

**Table 7:** Effects of human contact and parity on the reproductive performance of sows. Means and standard error of the difference (s.e.d.) for main effects are presented.

Measurement	Human Contact		Parity			*p*-Value		
	Positive	Control	Younger	Older	s.e.d.	Human Contact	Parity	Human Contact × Parity
Farrowing rate	0.83	0.79	0.82	0.81	0.038	0.40	0.78	0.66
Number of piglets born alive	12	12	12	12	0.41	0.71	0.57	0.53
Number of stillborn piglets	1.3	1.2	1.0	1.4	0.14	0.46	0.013	0.99
Number of mummified piglets	0.17	0.15	0.14	0.18	0.042	0.65	0.31	0.23
Number of piglets weaned	9.3	9.8	9.7	9.5	0.25	0.07	0.52	0.37

## Data Availability

The data presented in this study are available on request from the corresponding author.
